# Large effect of phosphate-solubilizing bacteria on the growth and gene expression of *Salix* spp. at low phosphorus levels

**DOI:** 10.3389/fpls.2023.1218617

**Published:** 2023-08-29

**Authors:** Piotr Koczorski, Bliss Ursula Furtado, Christel Baum, Martin Weih, Pär Ingvarsson, Piotr Hulisz, Katarzyna Hrynkiewicz

**Affiliations:** ^1^ Department of Microbiology, Faculty of Biological and Veterinary Sciences, Nicolaus Copernicus University, Torun, Poland; ^2^ Soil Science, Faculty of Agricultural and Environmental Sciences, University of Rostock, Rostock, Germany; ^3^ Department of Crop Production Ecology, Swedish University of Agricultural Sciences, Uppsala, Sweden; ^4^ Linnean Centre for Plant Biology, Department of Plant Biology, Uppsala BioCenter, Swedish University of Agricultural Science, Uppsala, Sweden; ^5^ Department of Soil Science and Landscape Management, Faculty of Earth Sciences and Spatial Management, Nicolaus Copernicus University, Torun, Poland

**Keywords:** transcriptome analysis, willow, phosphate solubilization, phosphate solubilizing microorganism, differential gene expression

## Abstract

Phosphorus is one of the most important nutrients required for plant growth and development. However, owing to its low availability in the soil, phosphorus is also one of the most difficult elements for plants to acquire. Phosphorus released into the soil from bedrock quickly becomes unavailable to plants, forming poorly soluble complexes. Phosphate-solubilizing bacteria (PSB) can solubilize unavailable phosphorus-containing compounds into forms in which phosphorus is readily available, thus promoting plant growth. In this study, two willow species, *Salix dasyclados* cv. Loden and *Salix schwerinii* × *Salix viminalis* cv. Tora, were inoculated with two selected bacterial strains, *Pantoea agglomerans* and *Paenibacillus* spp., to evaluate the plant growth parameters and changes in gene expression in the presence of different concentrations of tricalcium phosphate: 0 mM (NP), 1 mM (LP), and 2 mM (HP). Inoculation with PSB increased root, shoot and leaf biomass, and for the HP treatment, significant changes in growth patterns were observed. However, the growth responses to plant treatments tested depended on the willow species. Analysis of the leaf transcriptomes of the phosphate-solubilizing bacterium-inoculated plants showed a large variation in gene expression between the two willow species. For the Tora willow species, upregulation of genes was observed, particularly for those involved in pathways related to photosynthesis, and this effect was strongly influenced by bacterial phosphate solubilization. The Loden willow species was characterized by a general downregulation of genes involved in pathway activity that included ion transport, transcription regulation and chromosomes. The results obtained in this study provide an improved understanding of the dynamics of *Salix* growth and gene expression under the influence of PSB, contributing to an increase in yield and phosphorus-use efficiency.

## Introduction

Phosphorus is a key element required for proper plant development, and its deficiency can lead to disrupted shoot growth, delayed plant maturation, reduced plant resistance to pathogens or reduced leaf area and number (Prabhu et al., 2019). Furthermore, phosphorus in the soil is nonrenewable, and research in recent years confirms that the demand for this element is continually increasing (Bindraban et al., 2020). Modern artificial fertilizers contain phosphorus most often in the form of rock phosphate or superphosphate, the sources of which are limited worldwide, and their increasing price is becoming a serious problem for farmers wishing to use this type of fertilizer. In the soil, phosphorus compounds unavailable to plants can exist in two forms, namely, inorganic form and organic form, and only mineralization and solubilization processes can result in these phosphorus forms becoming available to plants (Richardson and Simpson, 2011). Soil phosphorus availability is impacted by pH (Penn and Camberato, 2019). In general, two phosphorus solubility maxima are observed at approximately pH levels of 4.5 and 6.5, which are associated with the lowest degree of phosphorus fixation by Ca, Al, and Fe minerals (Barrow, 2017; Penn and Camberato, 2019). In acidic soils, the dominant forms of inorganic phosphorus are aluminum (AlPO_4_-2H_2_O) or iron (FePO_4_-2H_2_O) compounds (Kalayu, 2019; Zhang B. et al., 2019). In alkaline soils, low-soluble and indirectly plant-available calcium compounds (dicalcium phosphate (DCP), tricalcium phosphate (TCP), etc.) are most often found (Peng et al., 2021). In these form, phosphorus can be leached from soils and ends up in groundwater and lakes until it reaches the bottom of the oceans where it is unrecoverable (Filippelli, 2008; Li et al., 2018).

Phosphate-solubilizing microorganisms (PSMs) can play a key role in the conversion of phosphorus compounds into bioavailable forms for plant uptake. Interest in this particular group of microorganisms and in the possibilities for their practical use in agriculture and forestry has been particularly important in recent years due to climate change, soil contamination by excessive use of fertilizers, drought and overexploitation of agricultural land (Tian et al., 2021). Knowledge of PSMs is progressively advancing by scientists from all over the world. A key action that would allow full use of PSMs in practice is the selection of strains with the highest efficiency, i.e., the ability to solubilize phosphorus compounds. PSMs can enhance phosphorus solubilization directly, e.g., through proton efflux, synthesis of acid and alkaline phosphatases or chelation of iron ions (*via* siderophores) (Tian et al., 2021). PSMs can also indirectly improve the ability of plants to take up nutrients (including phosphorus) through phytostimulation (synthesis of indole-acetic acid (IAA) or ACC deaminase) or the production of hydrolytic enzymes (Richardson and Simpson, 2011). Microorganisms in this group can promote the development of the plant root system and increase uptake efficiency of other key nutrients needed by plants (Bargaz et al., 2021). For this reason, PSMs are used as key components of engineered biofertilizers in modern agriculture, for example, in wheat, maize, rice or fruit trees such as apple trees (Kurek et al., 2013; Viruel et al., 2014; Liu et al., 2019; Kour et al., 2020; Gomez-Ramirez and Uribe-Velez, 2021.

We chose two *Salix* species that are commonly grown as short-rotation coppices in Europe as a renewable energy source as model plant species. The two *Salix* species, Loden and Tora, are characterized by substantial morphological differences. Most of the biomass of Loden willow species is allocated to the leaves, while that of Tora is mainly allocated to the shoots (Hoeber et al., 2018). In general, *Salix* species are also considered phosphorus efficient, suggesting that microorganisms associated with these plant species help promote their ability to solubilize phosphorus and maintain stable levels in the soil, making them the perfect model plant species for both investigation of microbial diversity and phosphorus-focused research.

Currently, in terms of phosphorus, most attention is given to understanding plant responses to phosphorus deficiency alone, without no consideration of the influence of microorganisms on this process (Ren et al., 2018; Mo et al., 2019; Wang et al., 2019, Zhang Y. et al., 2019; Sun et al., 2021). Analyzing the transcriptome of a plant inoculated with PSMs can help provide answers on the extent to which PSMs affect or influence their plant host. Under phosphorus-deficiency stress, plants activate gene expression cascades responsible for the synthesis of auxins, abscisic acid, jasmonic acid, salicylic acid and ethylene (Sun et al., 2016). Researchers have investigated the interactions of plants with arbuscular fungi and the effects of these interactions on the plant transcriptome (Liu et al., 2020; Ray et al., 2021). Fungi are responsible for activating genes responsible for auxin synthesis and responses to nitrogen or phosphorus deficiency (Ludwig-Müller, 2015). In terms of phosphorus metabolism, fungi are capable of activating genes responsible for the synthesis of phosphatases or genes related to the transport of phosphorus in plant tissues (Ray et al., 2021). However, there is a large gap in knowledge, as little attention has been given to the effect of bacteria on plants under phosphorus-deficient conditions. Soni et al. (2021) and his team examined the effect of the bacterium *Paenibacillus polymyxa* on the tobacco transcriptome. The researchers observed that in addition to activating genes responsible for promoting plant growth, bacteria can influence the transcription of many genes responsible for phosphorus transport (pstA, pstB, pstC, pstS, phnD or phnE) within the plant. However, this was the only publication we could find on the subject. A more in-depth understanding of the effects of bacteria on the plant transcriptome will allow a more accurate determination of their positive effects on plants, resulting in more effective bioinoculants in the future.

This study aimed to assess the effect of inoculation with PSB strains on the growth and gene expression dynamics in the leaves of two *Salix* species grown under three different phosphate concentrations. Therefore, our research involved screening from a pool of 64 PSB strains obtained in our previous study (Koczorski et al. (2022)) and selecting strains with the highest efficiency for solubilization of TCP and DCP for *in vitro* experiments. Second, evaluation of their potential to promote plant growth under conditions of phosphorus deficiency in the media was not only determined based on plant growth parameters but also characterized by the level of gene expression changes during plant inoculation with the PSB strains. We hypothesize that PSB strains not only can solubilize phosphate in the substrate but also can regulate phosphorus-related pathways in plants as well as stimulate plant growth and development. We speculate that at the initial stages of *Salix* growth, the effect of PSB (PSB) will be noticeable at the transcriptomic level in the leaf tissue.

## Materials and methods

### Phosphate-solubilizing bacterial strain collection

The PSB strains used in this experiment are part of a collection reported in our previous study (Koczorski et al., 2022). The bacteria were isolated (autumn 2018) from the roots and rhizosphere soil of two willow species: ‘Tora’ (Svalöf-Weibull (SW species No. 910007, *S. schwerinii* × *S. viminalis*) and ‘Loden’ (SW 890129, *S. dasyclados*). These willow trees were grown at two test sites established in 2014 as part of the ECOLINK project in Uppsala (Sweden) and Rostock (Germany) (Hoeber et al., 2018; Koczorski et al., 2021b). The selection of PSB was performed using three selection media containing calcium triphosphate (National Botanical Research Institute’s phosphate growth medium (NBRIP) and Pikovskaya (PVK)) or calcium diphosphate (DCP) as phosphorus sources (Koczorski et al., 2021a). The bacteria that showed the ability to solubilize phosphorus (presence of halo zones) were classified into the PSB group and identified based on their 16S rRNA sequences (OP102593-OP102680) (Koczorski et al., 2022).

### Selection of phosphate-solubilizing bacterial strains with the highest phosphorus solubilization efficiency

In the present study, a total of 64 bacterial strains from the PSB collection belonging to the species *Pseudomonas*, *Bacillus*, *Erwinia*, *Serratia*, *Paenibacillus* or *Burkholderia* (Koczorski et al., 2022) were screened. Solid NBRIP and DCP media were used to select the phosphate-solubilizing bacterial strains with the highest efficiency for phosphorus solubilization [the composition of the media is given in (Koczorski et al., 2021a)]. The bacteria were spot inoculated onto media (4 strains per Petri plate), and there were three replicates. The bacteria were cultured in the dark at 28°C, and the diameter of the halo zone was measured after 1, 5 and 10 days of culture. The results obtained were statistically analyzed; the 10 most efficient phosphate-solubilizing bacterial strains were selected and used for further analysis. The 10 selected bacteria did not include strains that were pathogenic to humans and plants, despite their high activity (data verified based on published literature).

The ten phosphate-solubilizing bacterial strains selected in the previous step were tested in liquid NBRIP media. NBRIP medium was chosen since plants cannot efficiently solubilize TCP on their own (Ticconi and Abel, 2004). In this part of the experiment, three different phosphorus concentrations were used, namely, 0 mM (NP), 1.0 mM (0.3129 g/L TCP) (LP) and 2.0 mM (0.6259 g/L TCP) (HP), and noninoculated media with the same variants were maintained as controls. The concentration of available phosphorus in the liquid NBRIP medium for all 10 strains was determined using the spectrophotometric molybdenum blue method (30 samples in total) (Nagul et al., 2015). Based on a statistical analysis, the 2 most effective PSB strains, *Paenibacillus* spp. and *Pantoea agglomerans*, were selected and used to inoculate the plants in the pot experiment.

### Inoculation of two willow species with PSB in conjunction with varying phosphate contents

Pots were divided into two compartments using membranes and filled with sterile sand (pH around 7). Loden (SW 890129, *S. dasyclados*) and Tora (SW 910007, *S. schwerinii* × *S. viminalis*) willow cuttings with two or more nodes were inserted into the pots such that there were two cuttings per pot (total 6 plants per variant). To minimize the effect of phosphorus sorption in the soil, quartz sand was used as a substrate. The pot experiment was designed to include three phosphorus concentrations and two willow species for inoculation treatments (control (Ctr), bacterium 1 (*Pantoea agglomerans* accession number: OP102615) and bacterium 2 (*Paenibacillus* spp. accession number: OP102612). Both bacteria were previously isolated from surface sterilized roots of Tora species. Prior to inoculation bacteria were cultivated on R2A medium (BD Difco, USA) for 2 days in 28°C then diluted to 0.7 OD600 in sterile water right before inoculation. The temperature in the room was set to 22°C with a photoperiod comprising 10 hours of light and 14 hours of darkness. After the cuttings acclimated for 7 days, with the exception of the control plants, the pots were inoculated with 10 ml of bacteria inoculum. The plants were watered thrice a week with a fertilizer solution with one of three different phosphorus concentrations (NP, LP, HP) for 1 month. We aimed to reach the final phosphorus concentrations (0.3129 g/L for LP and 0.6259 g/L for HP) in the first week of the experiment. After the plants were cultivated for 5 weeks, we harvested them and measured the length of their roots and shoots, along with measuring the fresh weights of their roots, shoots and leaves. In addition, the total phosphorus content in leaves at the end of the experiment was examined using soil fractionation method (Negassa et al., 2010) and via inductively coupled plasma–optical emission spectrometry (ICP–OES). The available phosphorus in soil substrate was measured using the spectrophotometric molybdenum blue method after sample extraction with 1% citric acid solution (Van Reeuwijk, 2002) for all the analyzed variants in the experiment (180 samples in total). A portion of the leaves obtained at harvest was flash frozen in liquid nitrogen and stored at -80°C.

### Statistical analysis

The plant growth parameters (leaf weight, shoot weight, root weight, shoot length, root length) and phosphorus concentrations in leaves and soil were analyzed using STATISTICA v.13.3.721 (StatSoft, Poland). As the results obtained were not normally distributed, the Kruskal−Wallis test was used to determine significant differences. Dunn’s *post hoc* test was performed to determine differences between inoculation variants, different phosphorus concentrations and the willow genotypes used. The data were analyzed using 3-way ANOVA, with soil phosphorus concentration (3 levels), *Salix* species (2 levels) and bacterial inoculation (3 levels) included as factors, and their interactions were also evaluated. The response variables included all the growth parameters and phosphorus concentrations in the soil and plant leaves.

### RNA isolation

Total RNA was extracted from 100 mg of leaf tissue and crushed in sterile mortars filled with liquid nitrogen. RNA extraction was performed according to the protocol of Chomczynski and Sacchi (2006), with slight modifications. A total of 1000 µl of TRIzol reagent (Life Technologies, Poland) was added to the crushed leaf material, which was then incubated for 5 minutes at room temperature. Then, 300 µl of chloroform was added, after which the material was incubated for 15 minutes and then centrifuged at 12000 rpm for 15 minutes. A mixture of 0.8 M sodium chloride, 1.2 M sodium citrate and 200 µl of isopropanol was added to the upper layer of the supernatant, after which the mixture was centrifuged. The resulting pellet was washed twice with 75% ethanol. The RNA pellet was then resuspended in RNAse-free water (50 µl) and stored at -80°C. The RNA samples were outsourced to the company Novogene (Great Britain) for library preparation and transcriptome analysis.

### Transcriptome analysis

For transcriptome analysis each variant of the experiment was represented by three samples. Messenger RNA was purified using poly-T oligo-attached magnetic beads. The first strand cDNA was synthesized using random hexamer primers, followed by the second strand cDNA synthesis using either dUTP for directional library or dTTP for non-directional library. The library was checked with Qubit and real-time PCR for quantification and bioanalyzer for size distribution detection. Quantified libraries will be pooled and sequenced on Illumina platforms, according to effective library concentration and data amount. The total RNA was sequenced using the Illumina platform in conjunction with P5 (P5-AATGATACGGCGACCACCGAGA (5’-3’) and P5’-TTACTATGCCGCTGGTGGCTCT (3’-5’) and P7 adapters (CGTATGCCGTCTTCTGCTTG-P7’ (5’-3’) and CATACGGCAGAAGACGAAC-P7 (3’-5’). After the removal of reads that were contaminated with adapter sequences, that included unknown nucleotides that constituted more than 10% of either read (N > 10%) and that were of low quality (base quality < 5) and constituted more than 50% of the sequence, the genome of *Salix viminalis* retrieved from the European Nucleotide Archive (ENA) was used as a reference for mapping using HISAT2 (Almeida et al., 2020). Assembly of the sequences was performed using StringTie software with transcripts of class code type ‘u’. Quantification was performed using featureCounts, and a differential analysis was performed using DESeq2 and edgeR with the following parameters: |log2(fold-change)| >= 1 &padj<= 0.05. Finally, enrichment analysis was performed using clusterProfiler with a padj< 0.05.

## Results

### Selection of bacterial strains with the highest efficiency of phosphorus solubilization

**Figure 1:**
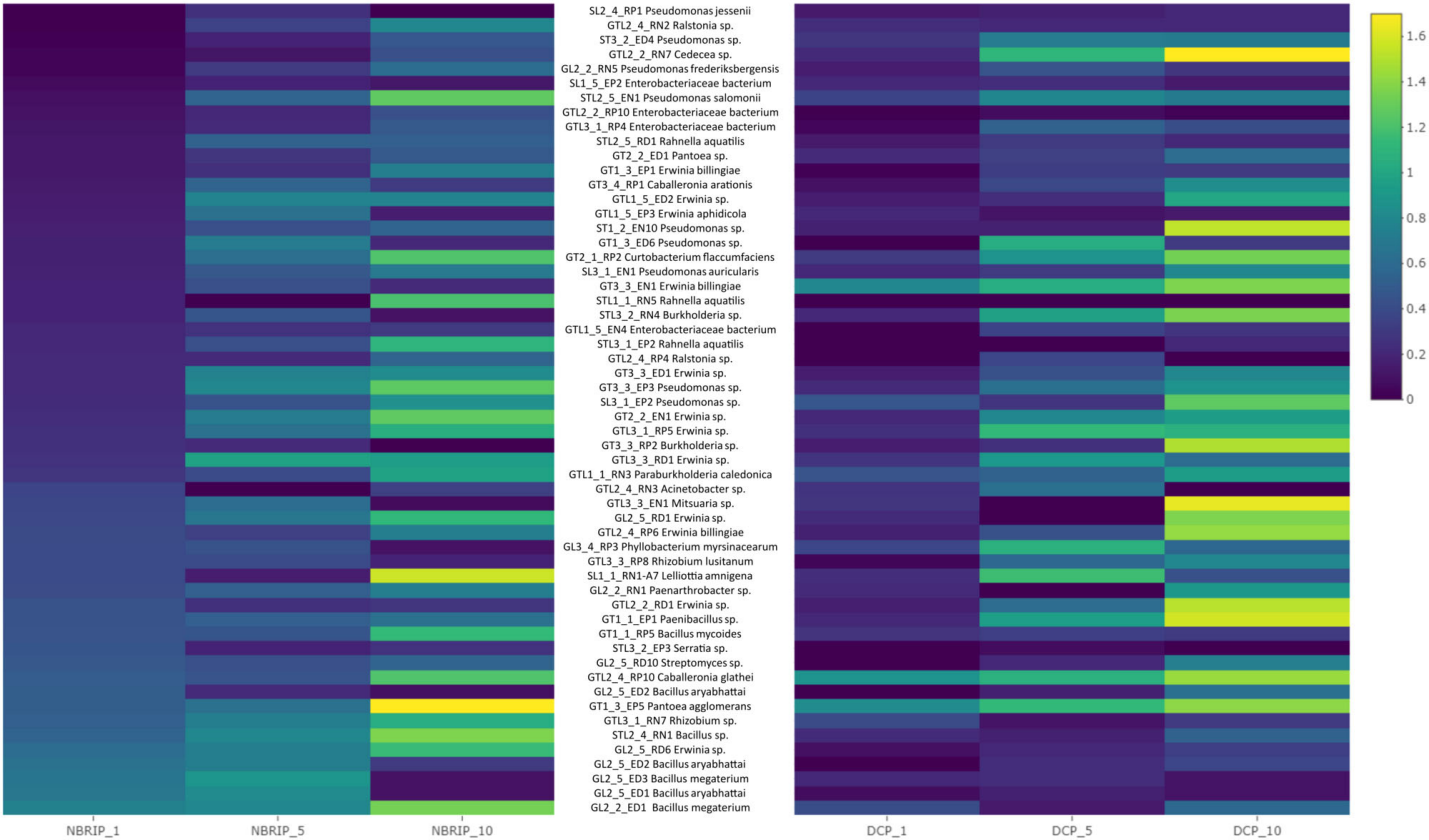
Heatmap of bacteria isolates ordered according to their phosphorus solubilization efficiency after 1 day of growth (from down to top). Activity was measured using solubilization index (from halo zone diameter we subtracted colony diameter). Measurements were taken after day 1, 5 and 10 from two selective media DCP and TCP. Blue colour indicates low solubilization index, while yellow high solubilization index.

The solubilization indices obtained from the experiment (given in the range from 0 to 1.6) are presented in the form of a heatmap where for each experimental variant (growth medium: DCP and NBRIP, strain: 64 in total and time of cultivation: 1, 5 and 10 days), the values are indicated based on color differences (from blue to yellow) (**Figures 1A, B**). The values in **Figure 1** are sorted according to the phosphorus solubilization efficiency measured on the NBRIP medium after 1 day of bacterial cultivation as the main criterion for the selection of microorganisms for further studies.

**Figure 2:**
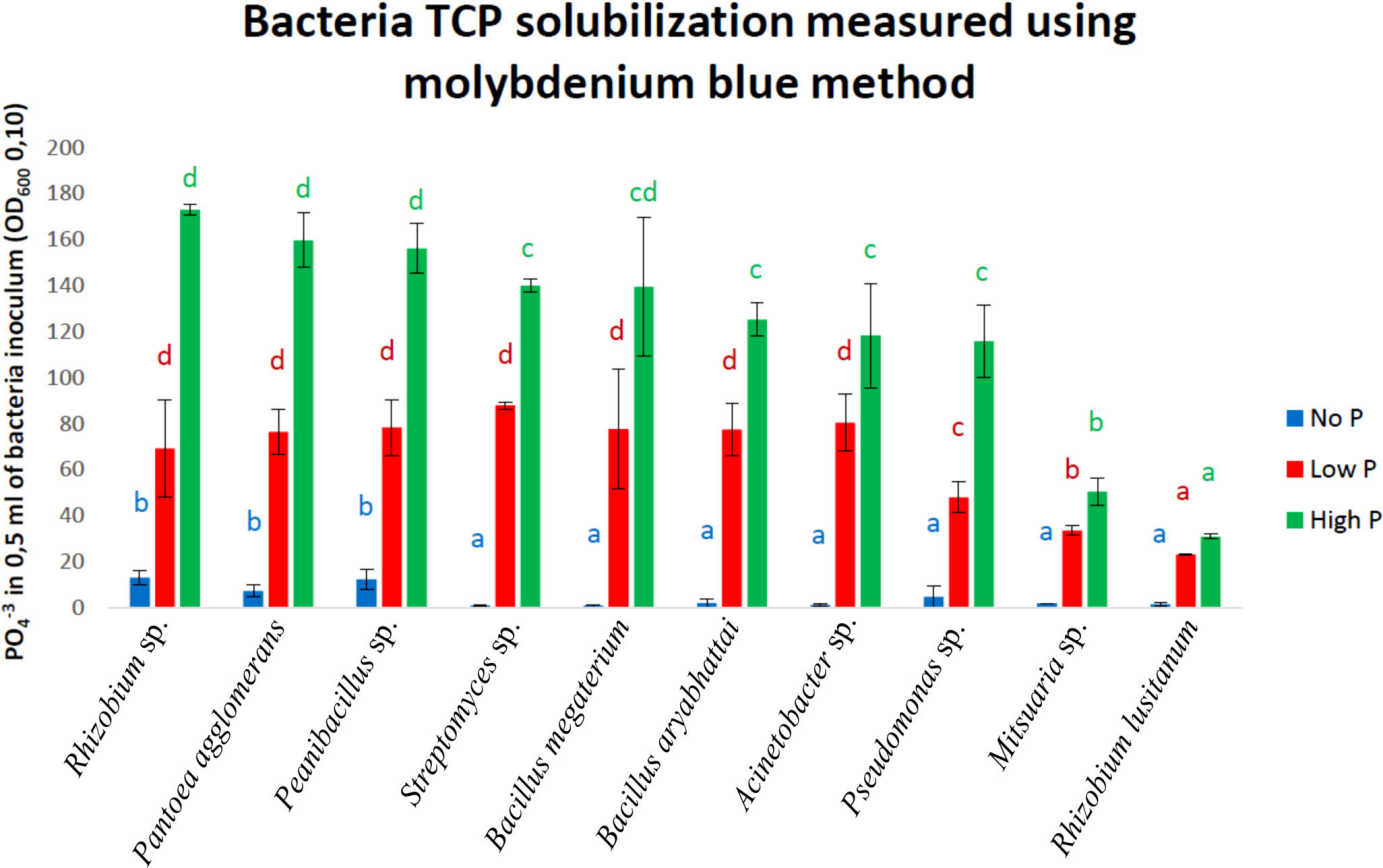
Phosphorus solubilization level represented by presence of PO_4_^-3^ in 0,5 ml of bacteria inoculum (OD_600_ 0.10) in liquid TCP medium. Media were supplemented with 3 different phosphorus concentrations: NoP – blue, LowP – red, HighP – green. Letters indicate significant differences between bacteria for each phosphorus variant.

Of the 64 strains tested, 15 with the strongest ability to solubilize phosphorus on DCP and NBRIP solid media were selected. In this group, we observed a predominance of species of the genus *Bacillus* (7 out of 15: *Bacillus megaterium*, *Bacillus aryabhattai*, *Bacillus* spp. and *Bacillus mycoides*); *Erwinia* (2 out of 15: *Erwinia* spp.); and single strains of *Rhizobium* spp., *Streptomyces* spp., *Pantoea agglomerans*, *Paenibacillus* spp., *Caballeronia glathei* and *Serratia* spp. The strains with the highest activity on NBRIP media after 10 days of incubation belonged to *Bacillus megaterium*, *Erwinia* spp., *Bacillus* spp. and *Bacillus aryabhattai*. The second DCP medium showed a slightly different distribution of strains in terms of their activity. Among the 15 strains previously selected (based on the NBRIP medium), only four displayed high activity for phosphorus solubilization of DCP: *Caballeronia glathei*, *Bacillus aryabhattai*, *Pantoea agglomerans* and *Paenibacillus* spp. Notably, the strains with the highest activity on the DCP medium included *Pseudomonas frederiksbergensis*, *Mitsuaria* spp., *Paenibacillus* spp. and *Pseudomonas* spp. Among the group of strains, *Bacillus megaterium*, *Bacillus aryabhattai*, *Erwinia* spp. and *Caballeronia glathei* were excluded from further study, as they were previously reported to be potential plant pathogens (Abdel-Monaim et al., 2012; Narsing Rao et al., 2019, Dobritsa and Samadpour, 2019, Parcey et al., 2022). A more detailed quantitative determination of bacterial phosphorus solubilization efficiency in liquid media (NBRIP) with different phosphorus contents showed that *Rhizobium* spp., *Pantoea agglomerans*, and *Paenibacillus* spp. were most efficient at solubilizing phosphorus compounds (**Figure 2**). Among the strains with significantly lower activity were *Streptomyces* spp., *Bacillus megaterium*, *Bacillus aryabhattai*, *Acinetobacter* spp. and *Pseudomonas* spp., while the *Mitsuaria* spp. and *Rhizobium lusitanum* strains displayed the lowest activity (**Figure 2**). These last two strains were excluded from subsequent stages of the study. *Rhizobium* spp. emerged as members among the most active strains; however, this species is not a typical endophyte and was excluded from further stages of the study.

### Bacterial contribution to plant growth promotion under phosphorus-limited conditions

All experimental factors (3 phosphorus concentrations, 2 willow species and 2 bacterial inoculations) showed statistically significant effects on most plant growth parameters (**Table 1**; **Supplementary Figures 1, 2**). A slightly better plant growth stimulation effect was observed for *Paenibacillus* spp. than for the other species (B2). Significant differences were also observed in response to different concentrations of phosphorus in the soil substrate. The positive or negative effects of the bacteria were dependent on the parameters tested, and the direction of change varied between the willow species and the bacterial variants (e.g., significant inoculation x species interactions listed in **Table 1**). High phosphorus concentrations in the soil significantly increased root fresh weight and length, while a decrease in leaf fresh weight was observed (**Table 1**). Soil phosphorus effects on plant growth patterns varied between the two willow species (e.g., the significant species x factor interaction effects in **Table 1**). Changes in soil phosphorus concentration did not affect the phosphorus content in the leaves (**Supplementary Figure 3**). Only B1 showed a significant decrease in leaf phosphorus content for the LP and HP treatments compared to the NP treatment for both Loden and Tora (**Supplementary Figures 3A, B**).

**Table 1:** ANOVA results for the effects of bacterial Inoculation (B1, B2), soil phosphorus concentration (NP, LP, HP) and willow plant Species (Loden, Tora) on various plant growth traits.

	Root length			Shoot length			Wet weight of roots			Wet weight of leaf			Wet weight of shoot		
	MS effect	F	p-level	MS effect	F	p-level	MS effect	F	p-level	MS effect	F	p-level	MS effect	F	p-level
(1) Inoculation	145.42*	20.198*	0.00000*	1891.82*	99.578*	0.00000*	1.78164*	139.884*	0.00000*	30.4661*	130.063*	0.00000*	6.0201*	44.970*	0.00000*
(2) phosphorus concentration	92.89*	12.902*	0.00001*	81.30*	4.280*	0.01677*	2.27967*	178.986*	0.00000*	7.1424*	30.491*	0.00000*	1.7321*	12.939*	0.00001*
(3) Species	1068.67*	148.435*	0.00000*	264.36*	13.915*	0.00033*	2.41824*	189.865*	0.00000*	0.1916	0.818	0.368185	3.1836*	23.781*	0.00001*
(1) x (2)	163.31*	22.684*	0.00000*	280.32*	14.755*	0.00000*	0.17112*	13.435*	0.00000*	2.4937*	10.646*	0.00000*	1.0162*	7.591*	0.00003*
(1) x (3)	193.21*	26.836*	0.00000*	38.16	2.009	0.140142	0.31205*	24.500*	0.00000*	0.1297	0.554	0.576823	1.9774*	14.771*	0.00000*
(2) x (3)	154.13*	21.408*	0.00000*	315.16*	16.589*	0.00000*	1.43592*	112.740*	0.00000*	4.9392*	21.086*	0.00000*	0.0498	0.372	0.690559
(1) x (2) x (3)	122.62*	17.032*	0.00000*	357.90*	18.839*	0.00000*	0.56060*	44.015*	0.00000*	1.8046*	7.704*	0.00002*	1.1084*	8.280*	0.00001*
Error	7.20			19.00			0.01274			0.2342			0.1339		
Tukey test for unequal sample sizes:															
(1) Inoculation	Ctr	11.62 A		Ctr	21.16 A		Ctr	0.38 A		Ctr	1.12 A		Ctr	1.07 A	
	B1	12.38 A		B1	31.41 B		B1	0.58 B		B1	2.74 B		B1	1.55 B	
	B2	15.42 B		B2	35.16 C		B2	0.83 C		B2	2.69 B		B2	1.89 C	
(2) phosphorus concentration	N P	11.29 A		No P	30.62 B		No P	0.36 A		No P	2.67 C		No P	1.41 A	
	LP	14.19 B		Low P	27.64 A		Low P	0.57 B		Low P	2.08 B		Low P	1.35 A	
	HP	13.93 B		High P	29.47 AB		High P	0.86 C		High P	1.80 A		High P	1.76 B	
(3) Species	Loden	9.99 A		Loden	27.68 A		Loden	0.45 A		Loden	2.23 A		Loden	1.68 B	
	Tora	16.28 B		Tora	30.81 B		Tora	0.75 B		Tora	2.14 A		Tora	1.33 A	

*indicate significant difference.

### Transcriptome analysis of two willow species in response to inoculation and limited phosphorus concentrations

**Figure 3:**
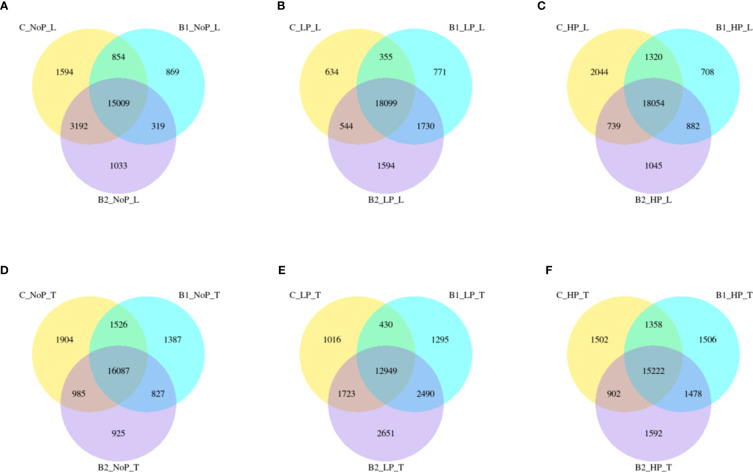
Venn diagrams representing common and unique genes expressed among inoculation variants. Loden – **(A, B)** and **(C)**; Tora – **(D, E)** and **(F)**;C – non inoculated control; B1, Bacteria 1; B2, Bacteria 2; NoP, No Phosphorus; LP, LowP; HP, HighP.

**Figure 4:**
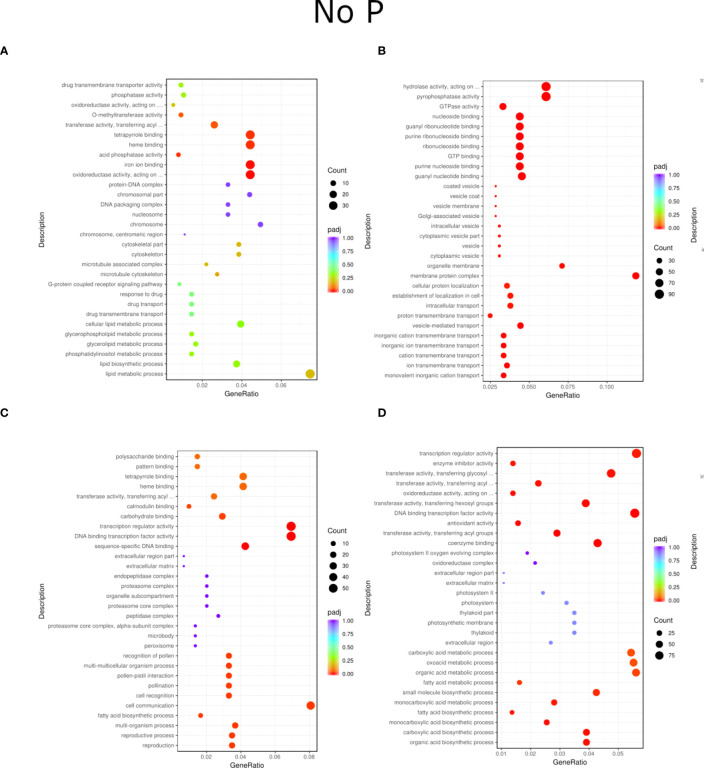
GO enrichment analysis scatter plots presenting the top 30 genes detected for the NP treatment of inoculation and phosphorus concentration for two willow species, Loden **(A, B)** and Tora **(C, D)**. The results are presented in pairs (B1 and B2) and are compared to the respective controls.

**Figure 5:**
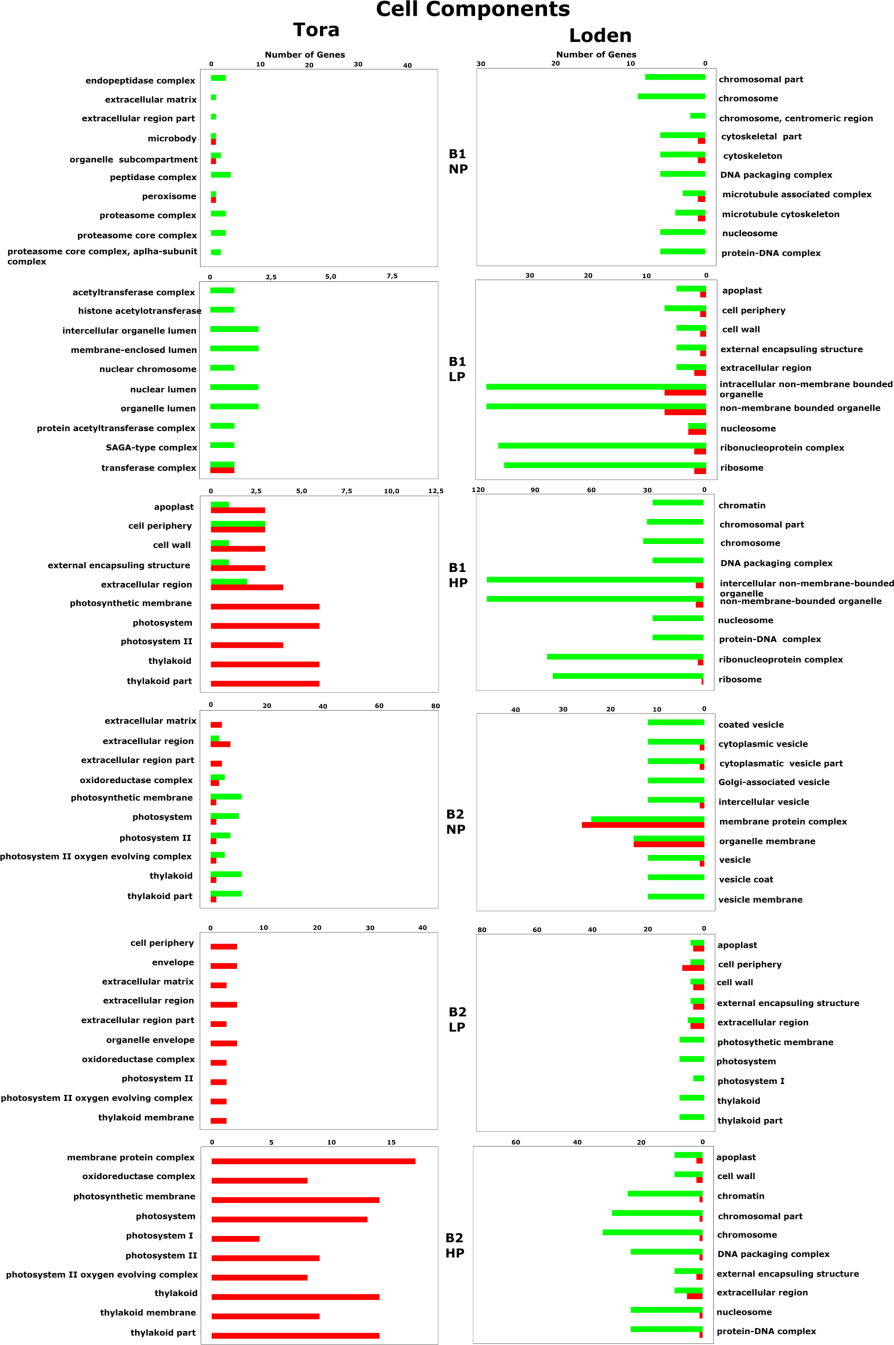
Cell component-associated gene expression regulation for all willow species, PSB and phosphorus concentrations. The red color indicates upregulation of genes, while the green color indicates downregulation.

**Figure 6:**
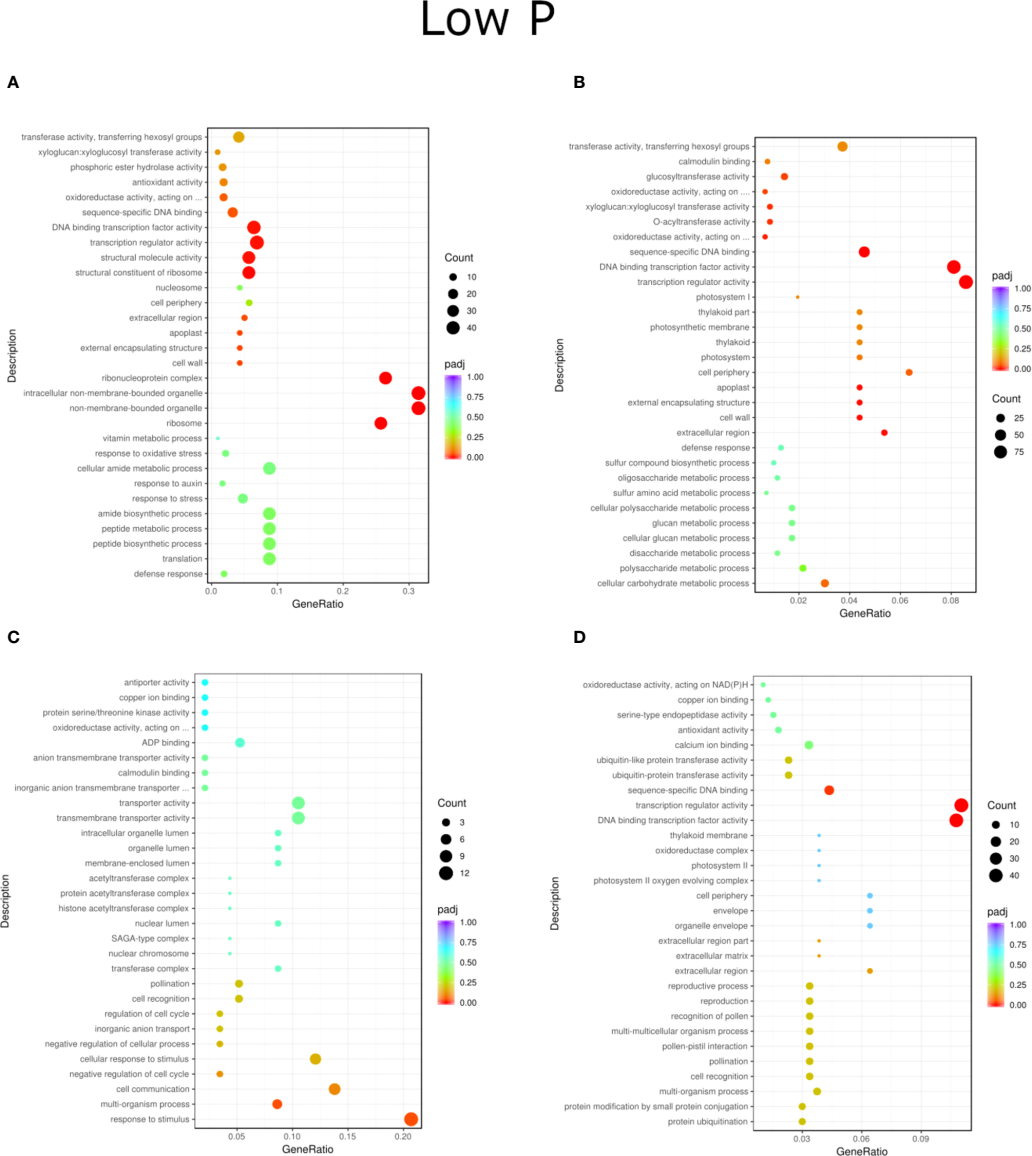
GO enrichment analysis scatter plots presenting the top 30 genes present for the LP treatment of inoculation and phosphorus concentration for two willow species, Loden **(A, B)** and Tora **(C, D)**. The results are presented in pairs (B1 and B2) and are compared to the respective controls.

**Figure 7:**
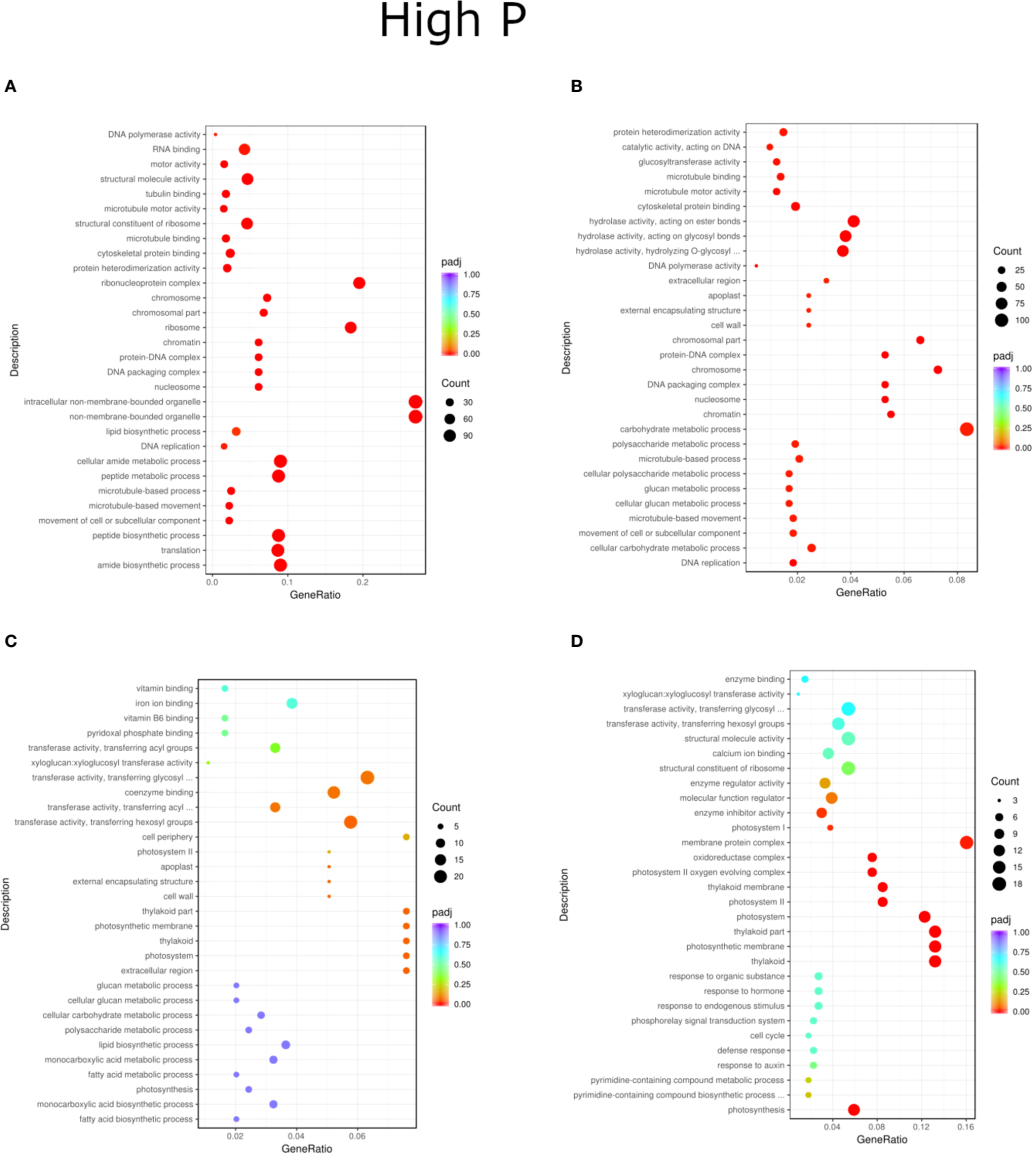
GO enrichment analysis scatter plots presenting the top 30 genes present for the HP treatment of inoculation and phosphorus concentration for two willow species, Loden **(A, B)** and Tora **(C, D)**. The results are presented in pairs (B1 and B2) and are compared to the respective controls.

Regardless of experiment variant 55% of reads from Loden species and 70% for Tora was mapped to reference genome used in this study. Additionally, PCA analysis was performed to check for intergroup differences between samples (**Supplementary Figure 4**). A transcriptome analysis of two willow species was performed to determine the effects of inoculation with the two selected PSB (B1 and B2) and different concentrations of phosphorus compounds (NP, LP and HP). The most distinctive difference was observed for plants inoculated with B2 of the Loden variety compared with the Ctr, where 2147 genes were downregulated and 2296 genes were upregulated. In addition, a similar trend was observed for both willow species in the HP and LP treatments: there was a greater number of genes that were upregulated compared to those in the NP treatment, the number of which ranged from 426 to 1920 genes (**Supplementary Figure 5**). No significant trend was observed in terms of downregulation of genes. The average numbers of all genes in Loden and Tora whose transcription patterns differed from those of the control variant were approximately 2500 and 1000, respectively. According to Venn diagrams (**Figures 3A–F**) for the Loden species, a pool of genes common to both the Ctr and B1 and B2 variants was lower in the NP (15009 genes) treatment compared to the LP (18099 genes) and HP (18054 genes) treatments (**Figures 3A–C**). In addition, a very low number of common genes between the B1 variants and the Ctr were observed in the LP treatment for the Loden (**Figure 4B**) and Tora (**Figure 3E**) variants, which were 355 and 430, respectively, the lowest values recorded. As shown in **Figures 4**–**6**, a Gene Ontology (GO) enrichment analysis was performed, and the 30 most differentially expressed genes associated with *p* values under 0.05 are presented for three phosphorus concentrations and two inoculation variants (bacteria B1 and B2 are compared to the corresponding non inoculated Ctr). Analyzing the effect of different phosphorus concentrations on the Loden willow species, we characterized the molecular functions in the NP treatments according to the presence of a large number of genes responsible for tetrapyrrole binding, haem binding, iron ion binding, GTP binding or ribonucleotide binding (**Figures 4A, B**). The LP treatment revealed a high number of genes responsible for the process of translation, and the HP treatment displayed a high number of genes associated with cytoskeleton formation and rearrangement (**Figures 5A, B**, **6A, B**). In the case of the inoculation effect, the Loden variants inoculated with B1 (rather than B2) demonstrated a high expression of genes involved in cell membrane structure, phosphatase activity and acid phosphatase activity (**Figures 4**, **6**, **7**). The variants inoculated with B2 were characterized by genes involved in hydrolase activity and pyrophosphatase activity and many genes related to cation and ion transport (**Figures 4**, **6**, **7**). For the LP treatment, significant changes in the molecular functions of plants inoculated with B1 and B2 were observed, although some similarities were detected. In response to this variant, a significant increase in the number of genes responsible for DNA binding and regulation of transcription and translation was observed (**Figures 6A, B**). However, enrichment of GO terms related to direct phosphorus solubilization was not observed. Similar to the NP treatment, a number of genes responsible for the biosynthesis and metabolism of peptides that make up cell membranes were noted. In the category of biological processes, the variants inoculated with B1 under LP revealed processes related to the stress response (especially oxidative stress) and the plant defense system (**Figure 6A**). In the HP treatment, a shift from transcription to translation and, in particular, to ribosome synthesis was observed for the genes (**Figures 6A, B**). Moreover, genes related to chromatin formation and the formation of ribonucleoprotein complexes were found. For the NP, LP and HP treatments, GO terms associated with cell membranes were detected among the differentially expressed genes. The plants inoculated with B2 for HP were also characterized by genes related to different types of hydrolases, which were not observed in response to B1.

For the Tora willow species under NP, a high frequency of genes responsible for transcription, DNA binding and organic acid metabolism was observed (**Figures 4C, D**). Plants under the LP treatments were characterized by genes associated with transport and pollination, whereas under HP, a high frequency of genes associated with chloroplasts, thylakoids and photosystems I and II was observed (**Figures 6C, D**, **7C, D**). For the effect of inoculation, B1-inoculated plants showed molecular functions belonging to tetrapyrrole and haem-binding activity and biological processes related to pollen recognition and formation and intercellular communication (**Figures 4C**, **6C**, **7C**). The plant inoculated with B2 was mainly characterized as expressing genes involved in metabolic processes and the biosynthesis of carboxylic acids, oxyacids and organic acids (**Figures 4D**, **6D**, **7D**). The Tora willow species under LP showed a change in gene expression and significant differences between the B1 and B2 variants (**Figures 6C, D**). In the case of the B1 variant, a high number of genes responsible for the stimulus response even at the cellular level was noted. A large number of genes responsible for transport also was detected. B2 under LP elicited the expression of a large number of genes responsible for transcription (**Figure 6D**). The he expression of genes related to pollen production, pollination and modification of various proteins was also evident, albeit with lower numbers. As with the LP treatment, little similarity was observed between B1 and B2 inoculation (with the exception of genes encoding transferases) (**Figures 6C, D**). Inoculation of plants with B1 resulted in high expression of genes responsible for transferase activity and coenzyme binding. A large number of genes related to the metabolic activity of various acids, glucan, lipids and carbohydrates also emerged. Inoculation with B2 increased the expression of genes involved in photosynthesis (thylakoid structure, photosystems I and II) and the synthesis of structural elements of plant cells. For each of the NP, LP and HP treatments, upregulation of a large number of genes responsible for the activity of different types of transferases was observed.

### Gene expression regulation

**Figure 8:**
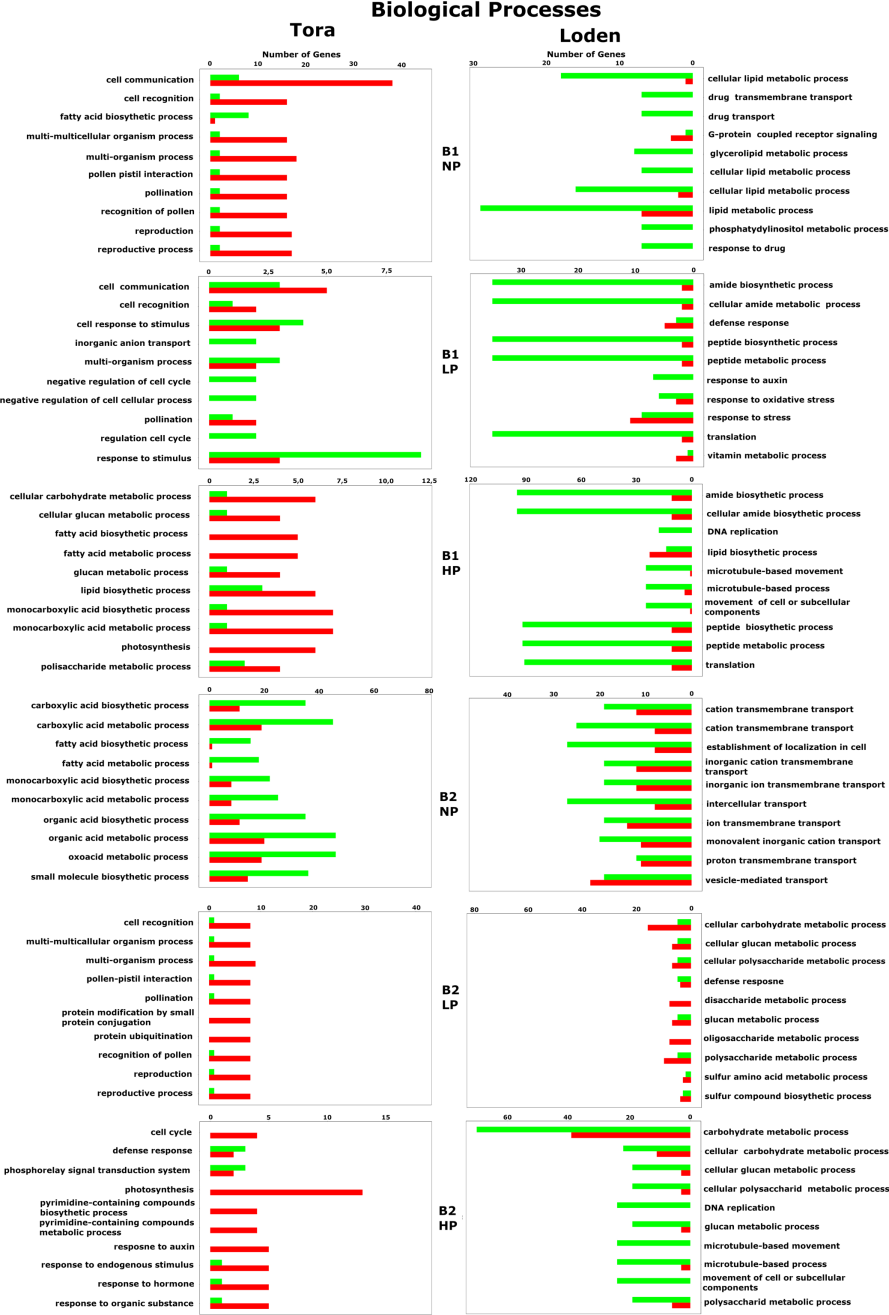
Biological process-associated gene expression regulation for all willow species, PSB and phosphorus concentrations. The red color indicates upregulation of genes, while the green color indicates downregulation.

**Figure 9:**
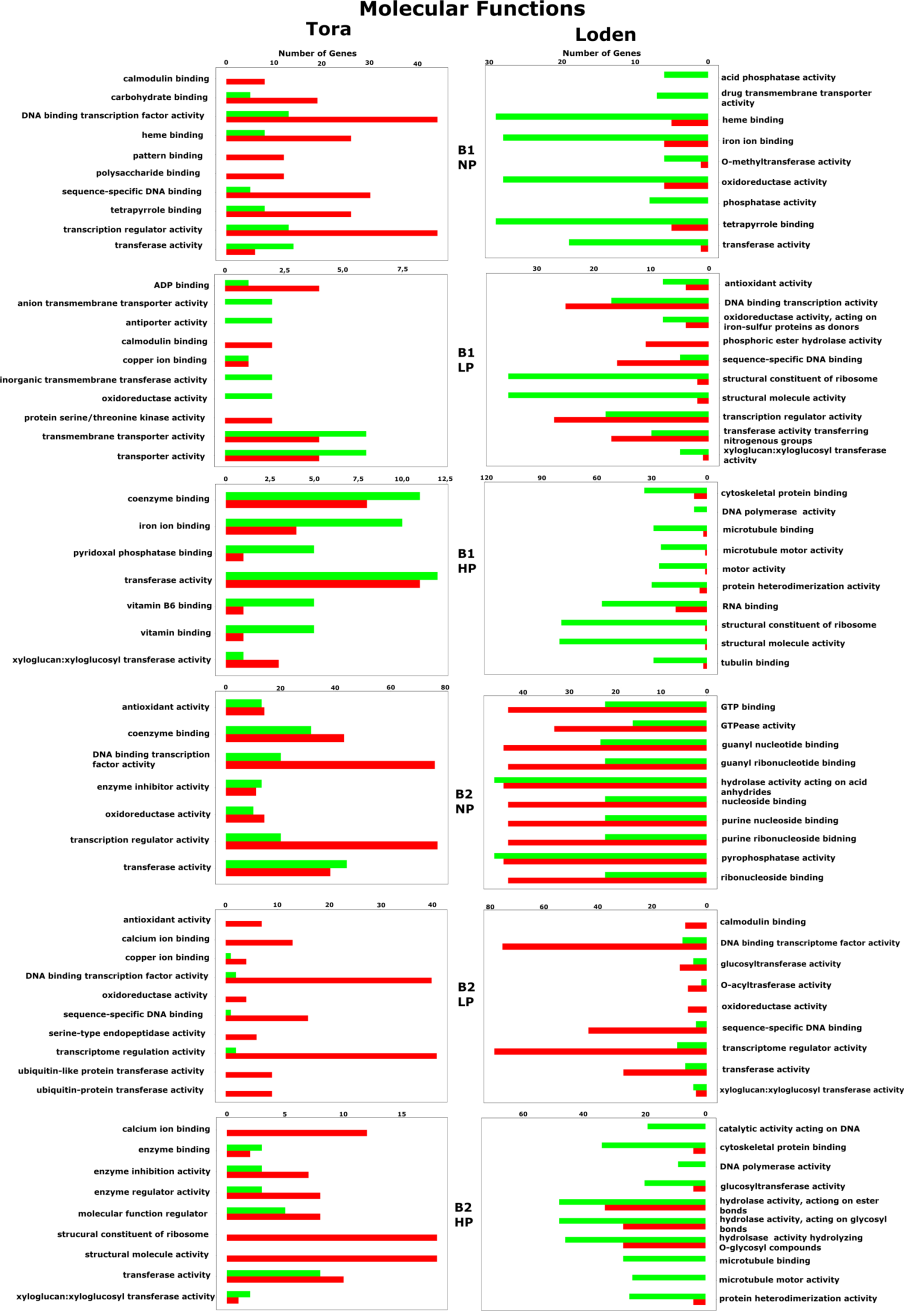
Molecular function-associated gene expression regulation for all willow species, PSB and phosphorus concentrations. The red color indicates upregulation of genes, while the green color indicates downregulation.

Among the 10 most frequently up- or downregulated genes found in each experimental variant, a general trend based on the willow species/species was observed in which genes for the Loden willow species were downregulated and genes for the Tora willow species were upregulated regardless of the experimental conditions tested (bacterial inoculant B1 or B2 and phosphorus concentration). This was particularly evident for genes associated with biological processes (**Figure 8**) and molecular functions (**Figure 9**).

Analysis of GO biological processes (**Figure 8**) revealed the occurrence of 86 different up- or downregulated genes across all factors tested in the experiment. Among these genes, 43 were specific only to Loden, and 37 were specific to Tora. There were also 6 genes that were either up- or downregulated in both plant species tested. These genes were responsible for the cellular carbohydrate metabolic process, glucan metabolic process, glucan metabolic process, defense response, response to auxin, and lipid biosynthetic process. In response to both inoculation variants (B1 and B2), 13 common genes were observed, mainly those related to the metabolism and biosynthesis of fatty acids, glucan, and carboxylic acids. A strong effect of phosphorus (NP, LP, HP) was observed for fatty acid and monocarboxylic acid biosynthesis and metabolism, for which downregulation was observed in the NP treatment and upregulation in LP and HP treatments.

In terms of the GO cellular components, 65 different genes were observed, of which 28 were specific only to Loden (mainly downregulation), and 27 were specific to Tora (mainly upregulation) (**Figure 5**). At 10, the pool of common genes for cellular components was larger than that for biological processes and included a large number of genes related to cellular elements involved in photosynthesis, i.e., thylakoids, photosystems I and II, the photosystem II oxygen-evolving complex and photosynthetic membranes. In response to different inoculants (B1 *vs*. B2), we observed the occurrence of 16 common genes, which were associated with terms related to chromosomes, the apoplast, DNA packaging or extracellular elements in addition to photosystem elements. For genes associated with cellular components, we also observed a strong effect of phosphorus on photosystems I and II, thylakoids, the photosystem II oxygen-evolving complex, and the oxidoreductase complex; moreover, the NP treatment downregulated the expression of these, in contrast to the LP and HP treatments, which upregulated the expression of these genes.

For GO molecular functions, our analysis revealed the presence of 73 genes, among which 37 were expressed only in Loden, and 27 were expression in Tora (**Figure 9**). The pool of common genes comprised 11 genes and included those mainly responsible for calmodulin, haem and calcium binding, transferase activity, transcription and DNA-binding processes. We observed that the gene pool shared by Loden and Tora was characterized by the lack of an apparent division between downregulated and upregulated genes. The pool of common genes that responded to both B1 and B2 totaled 12. The abovementioned group of genes was joined by microtubule-binding activity, DNA-binding transcription factor activity and copper ion binding. In the case of molecular functions, the influence of phosphorus on gene regulation was observed only in the case of coenzyme binding, where the HP treatment elicited upregulation, which contrasted with the NP treatment, which caused those genes to be downregulated. Additionally, downregulation of two genes related to phosphatase activity was characteristic for the Loden B1 NP treatment combination.

## Discussion

In an era of climate change and a growing human population on Earth, there is a challenge in providing humanity with adequate food and energy resources, which are critical for such rapid population growth. The increased use of phosphorus fertilizers in agriculture worldwide is causing many undesirable environmental changes and depleting the planet’s supply of this element (Filippelli, 2008; Alewell et al., 2020). The results of this experiment highlight the large effects of phosphorus provided by PSB in terms of altering growth patterns and gene expression, which manifested in the form of increased shoot growth for both willow species and general upregulation of genes for Tora and downregulation of them for Loden. The findings presented in this paper could constitute an important piece of the puzzle for developing effective bioinoculants containing PSB to increase the soil phosphorus use efficiency of plants.

### Phosphate-solubilizing bacterium selection

From a pool of 64 PSB, two strains with the highest TCP-solubilization potential were selected (based on two screening stages carried out on solid and liquid TCP media) to investigate the effects of inoculation on the growth of willow seedlings under different phosphorus concentrations. The willow plants were grown in a substrate supplemented with TCP (Ca^3^(PO^4^)^2^), which is not a readily available source of P, and the phosphate solubilization process carried out by the bacterium inoculants was the only source of available P, with the exceptions of strains *Bacillus aryabhattai* (GL2_5_ED2) and *Caballeronia glathei* (GTL2_4_RP10), which solubilized TCP and DCP equally well. *Bacillus* spp. are well-known and well-documented PSM and have been tested many times for their use as potential biofertilizers of crop plants (Tahir et al., 2018; Ahmad et al., 2019; Miljaković et al., 2020; Mosela et al., 2022). In the present experiment, among the 15 most effective strains that solubilized phosphates, *Bacillus* spp. also were predominant (7 out of 15). This study found that *Rhizobium* sp. bacteria solubilized phosphate compounds most effectively, which highlights their great potential not only for nitrogen fixation but also as free-living soil bacteria that can solubilize P. Recent literature has confirmed the high efficiency of *Rhizobium* applied to crop plants, but there are no reports of the role of this species in tree growth (Afzal and Bano, 2008; Jaybhay et al., 2017; Verma et al., 2020; Mir et al., 2021; Shome et al., 2022).

In the pot experiment, strains *Pantoea agglomerans* (B1) and *Paenibacillus* sp. (B2) that effectively solubilized phosphates in both LP and HP media (analyzed by the molybdenum blue method) were selected. *Pantoea agglomerans* is described as a plant growth-promoting rhizobacterium (PGPR) capable of auxin biosynthesis, ACC deaminase production, ammonia production, phosphorus solubilization and increasing plant tolerance to salt stress (Majumdar and Chakraborty, 2015; Cherif-Silini et al., 2019; Emami et al., 2021). Because willow trees do not constitute a food crop, the potential pathogenicity of willow to humans was not an issue (Mackiewicz et al., 2016). The second strain we selected was *Paenibacillus* sp., which is described in the literature as a bacterium capable of nitrogen fixation; phosphorus solubilization; stimulation of the plant defense system; and production of ammonia, HCN, IAA and siderophores (Kumari & Thakur, 2018; Hussain et al., 2020). Both bacterial species have been previously experimented with as bioinoculants of plant species such as barley (Canbolat et al., 2006), wheat (Hussain et al., 2020), sugarcane (Quecine et al., 2012), poplar (Vaitiekūnaitė et al., 2021) and palm trees (Saadouli et al., 2021).

### Effects of inoculation on willow growth

Inoculation with PSB and phosphorus treatments had significant effects on the growth patterns of the tested plants. However, it was difficult to determine whether the changes were caused by different soil phosphorus levels, bacterial inoculation, or a combination of both. Growth responses were different for Loden and Tora. For Loden, an increase in shoot thickness was found in response to higher soil P, while for Tora, the shoot length increased. In the NP treatment, the decrease in the leaf fresh weight could have been the result of phosphorus deficiency, because phosphorus deficiency has been shown to stimulate root allocation at the cost of leaf allocation (Ericsson, 1995). However, changes in phosphorus allocation to the roots or the leaves could also be related to changes in substrate pH, because increasing TCP concentrations may have increased the substrate pH. According to Walle et al. (2007), the optimum pH for willow growth is between 5 and 7.5, and the process of phosphorus solubilization by microorganisms is often associated with soil acidification (Liang et al., 2013). While alkaline phosphorus compounds are being formed, the pH becomes equilibrated by the process of microbial phosphate solubilization (Khan et al., 2014).

### Effects of increasing phosphorus concentration and phosphate-solubilizing bacterium inoculation on willow gene expression

Plant transcriptome studies are becoming an integral part of all analyses aimed at determining the impact of biotic or abiotic stresses on various biological processes, cellular components or molecular functions. There are a growing number of publications examining the transcriptomic responses of tree crops and the impact of factors such as drought (Pucholt et al., 2015; Jia et al., 2020; Xu et al., 2021), salinity (Yao et al., 2018; Zhou et al., 2020; Pang et al., 2022) and pest resistance (Wang et al., 2020). To date, however, there are no publications showing differences in gene expression under P-deficient conditions. Our results allowed us to determine the effects of phosphate-solubilizing bacterium inoculation with increasing concentrations of plant-available phosphate on the expression of genes related to the phosphorus stress response and phosphorus metabolism, mobilization and remobilization.

Our transcriptomic analysis showed high variability between the gene expression in the leaves of the two willow species tested (Loden *vs*. Tora), the inoculation variants (B1 and B2) and the phosphorus concentrations in the substrate (NP, LP, HP). The results showed that Loden exhibited approximately 1500 more differentially expressed genes than did the Tora willow species. As our previous study has shown, these species differ significantly in their morphology (Hoeber et al., 2017) and root-associated microbiome (Koczorski et al., 2021a; Koczorski et al., 2022). As reported by Hoeber et al. (2017), Tora is characterized by highly efficient biomass production (tall shoots) but a smaller leaf area. Conversely, Loden is characterized by a large leaf area but relatively low amount of biomass production. Moreover, under NP conditions, Loden was shown to express a low number of genes in common between both the B1 and B2 variants, suggesting that inoculation significantly affects gene activity, specifically under these conditions. According to previous studies, one of the plant responses to inoculation is increased activity of genes responsible for DNA replication and cell division (Camilios-Neto et al., 2014). In addition, the activity of genes related to response to stimuli, signaling, regulation of biological processes and metabolic processes were observed (Liu et al., 2012). In our analysis, similar gene activities in both the Loden and the Tora willow species, especially under LP conditions, were observed. Additionally, the expression of genes responsible for photosynthesis, transcription and translation in the LP and HP treatments was also observed. This was manifested by the activity of genes such as those involved in the glucan metabolic process, thylakoids, photosystems I and II, translation, DNA-binding translation factor activity, transcription regulation and sequence-specific DNA binding.

Phosphorus plays a very important role in the photosynthetic carbon reduction cycle, also known as the Calvin-Benson cycle, because the final product of this process is 3-phosphoglyceraldehyde (triose-P), which is then (already in the form of sucrose) transported to the cytosol of the cells or remains in the chloroplasts where it is converted into starch (Rychter and Rao, 2005). Among the previously mentioned genes are also those responsible for glucan metabolic processes. Glucan phosphatase plays an important role in the transient metabolism of starch in leaves, as glucan phosphatase is the main storage material and consists mainly of glucose polymers. During the diurnal cycle of photosynthesis, starch is deposited during the day to be used up at night to provide sufficient metabolites needed for plant growth (Meekins et al., 2016). Our results suggest that the bacteria present in the substrate significantly improved the availability of starch and thus had a positive effect on plant growth. For the Tora willow species inoculated with B1, activity of genes responsible for pollen recognition and formation (NP and LP treatments) was observed. The direct effect of inoculation on pollen formation has not yet been investigated, but according to a study by Lankinen et al. (2000), phosphorus has a positive effect on the quantity and quality of pollen formed, while abiotic stresses such as salinity do not affect this process. Among the variants tested, only the Loden willow species inoculated with B1 under LP showed the expression of genes directly related to stress and defense mechanisms. Studies conducted on *Arabidopsis thaliana* leaves under P-deficiency conditions showed that these plants exhibit high expression of genes involved in phosphatase synthesis and genes that encode ribonucleases and sulfolipid biosynthesis proteins that replace P, contributing to phosphorus remobilization (Müller et al., 2007). Similarly, we detected genes involved in ribonuclease activity and phosphoric ester hydrolase activity, which may indicate P-deficiency stress. The genes indicated above (except for those encoding ribonucleases) were also expressed at lower numbers in response to the other treatment combinations in this study, which may be related to the rapid growth of the plants at an early stage of development. As a woody plant species, willows are known for their rapid growth, and as research has indicated, phosphorus remobilization and phospholipid replacement by sulfur and galactolipids is a strategy employed to increase photosynthetic efficiency (Lambers et al., 2012).

### Gene expression regulation

The information obtained in this analysis largely overlaps with the results obtained from the GO enrichment analysis, providing additional information about genes that are upregulated and downregulated. The first and most prominent result of the analysis is the observed trend, in which the genes of the Loden willow species were downregulated and those of the Tora willow species were upregulated. As mentioned earlier, this may be a result of the difference in biomass allocation between the two willow species (Ericsson, 1995).

For the Tora willow species, a large effect of phosphorus on genes related to photosynthesis, thylakoids and starch metabolism in the form of upregulation of photosynthesis, photosystems I and II, thylakoid parts, carboxylic acid, carbohydrates, glucan metabolism and biosynthesis genes was found. All of the abovementioned genes are highly dependent on phosphorus because it is a component of ATP, NADPH, nucleic acids, sugar phosphates and phospholipids (Carstensen et al., 2018). The effect of phosphorus on carboxylic acids seems to be substantial due to the important role of these compounds as plant signalling molecules; also important are compounds like malate, which can be converted into NADH and NADPH, thus increasing the efficiency of photosynthesis (Mutz et al., 2013). Additionally, the presence of carboxylic acids has been confirmed in the apoplast, where activity related to the regulation of stomatal opening occurs (Meyer et al., 2010).

Among the downregulated genes for the Loden willow species, genes involved in lipid peptide metabolism and biosynthesis, nucleic acids, and ion and metabolite transfer were detected, while upregulation of genes involved in the defense mechanism and response to stress was also observed. Gene expression studies conducted on plants subjected to P-deficiency stress have indicated that the most common plant response to phosphorus deficiency is the downregulation of genes involved in ATP synthesis, translation, transport, carbohydrate synthesis and photosynthesis, which was observed for all the treatment combinations involving Loden (Bai et al., 2014; Chu et al., 2018). In addition, the upregulation of genes related to sulfur compounds was observed for the B2-inoculated Loden treatment combinations, indicating an attempt to replace phosphorus to increase phosphorus-use efficiency (Lambers et al., 2012). Notably, plants in the LP and HP treatments did not show signs of phosphorus deficiency, so the phenomena are related to species differences and biomass allocation rather than phosphorus itself.

## Conclusions

Inoculation of *Salix* spp. with PSB increased soil phosphorus uptake and stimulated plant growth, which was manifested through changes in the growth patterns of both willow species and changes in the regulation of gene expression at the transcriptional level. Significant species differences were observed between Loden and Tora in their responses to the two inoculation variants and the different phosphorus concentrations in the substrates, the differences of which were evident, e.g., an increase in shoot thickness for Loden in response to higher soil phosphorus and a greater shoot length for Tora under the same treatment conditions. Transcriptomic analysis showed that phosphate-solubilizing bacterium inoculation of the Tora willow species significantly affected transcription in the leaves, affecting the upregulation of most genes, especially those related to photosynthesis, which are highly influenced by phosphorus. A general reduction in gene transcription levels was observed in Loden, especially for genes involved in ion transport, transcriptional regulation and chromosomes.

## Data availability statement

The datasets presented in this study can be found in online repositories. The names of the repository/repositories and accession number(s) can be found in the article/**Supplementary Material**.

## Author contributions

PK participation in all analyses and preparation of initial draft, data processing and preparation of figures and tables, and statistical analysis. BF participation in all analyses and preparation of initial draft, review and editing of draft. CB pot experiment design, review, and editing of draft. MW review and editing of draft. PI substantive support during transcriptome data interpretation, review, and editing of draft. PH soil analyses, review, and editing of draft. KH experiment design and supervision, review and editing of draft. All authors contributed to the article and approved the submitted version.
