# Supplementary material for: Large effect of phosphate-solubilizing bacteria on the growth and gene expression of Salix spp. at low phosphorus levels
Source: Front Plant Sci. 2023 Aug 29;14:1218617. doi: 10.3389/fpls.2023.1218617 (PMC10495996; doi:10.3389/fpls.2023.1218617)
Supplement: Supplementary file 1 [file DataSheet_1.pdf]

Supplementary Material

Large effect of phosphate-solubilizing bacteria on the growth and gene expression of Salix spp. at low phosphorus levels

Piotr Koczorski, Bliss Ursula Furtado, Christel Baum, Martin Weih, Pär Ingvarsson, Piotr Hulisz and Katarzyna Hrynkiewicz

*** Correspondence:**
Katarzyna Hrynkiewicz; hrynk@umk.pl

Keywords: transcriptome analysis, willow, phosphate solubilization, phosphate-solubilizing microorganism, differential gene expression

Supplementary Material

Data availabilability

The reference genome of *Salix viminalis* was acquired from Almeida et al. 2020

Almeida, P., Proux-Wera, E., Churcher, A., Soler, L., Dainat, J., Pucholt, P., Nordlund, J., Martin, T., Rönnberg-Wästljung, A.C., Nystedt, B. and Berlin, S., 2020. Genome assembly of the basket willow, Salix viminalis, reveals earliest stages of sex chromosome expansion. *BMC biology*, *18*(1), pp.1-18.

Bacteria used in the study can be found in supplementary materials of Koczorski et al., 2022

Koczorski, P., Furtado, B. U., Gołębiewski, M., Hulisz, P., Thiem, D., Baum, C., et al. (2022). Mixed growth of Salix species can promote phosphate-solubilizing bacteria in the roots and rhizosphere. Front. Microbiol. 13. doi: 10.3389/fmicb.2022.1006722

Transcriptome data is available at https://www.ncbi.nlm. nih.gov/bioproject, PRJNA967604.

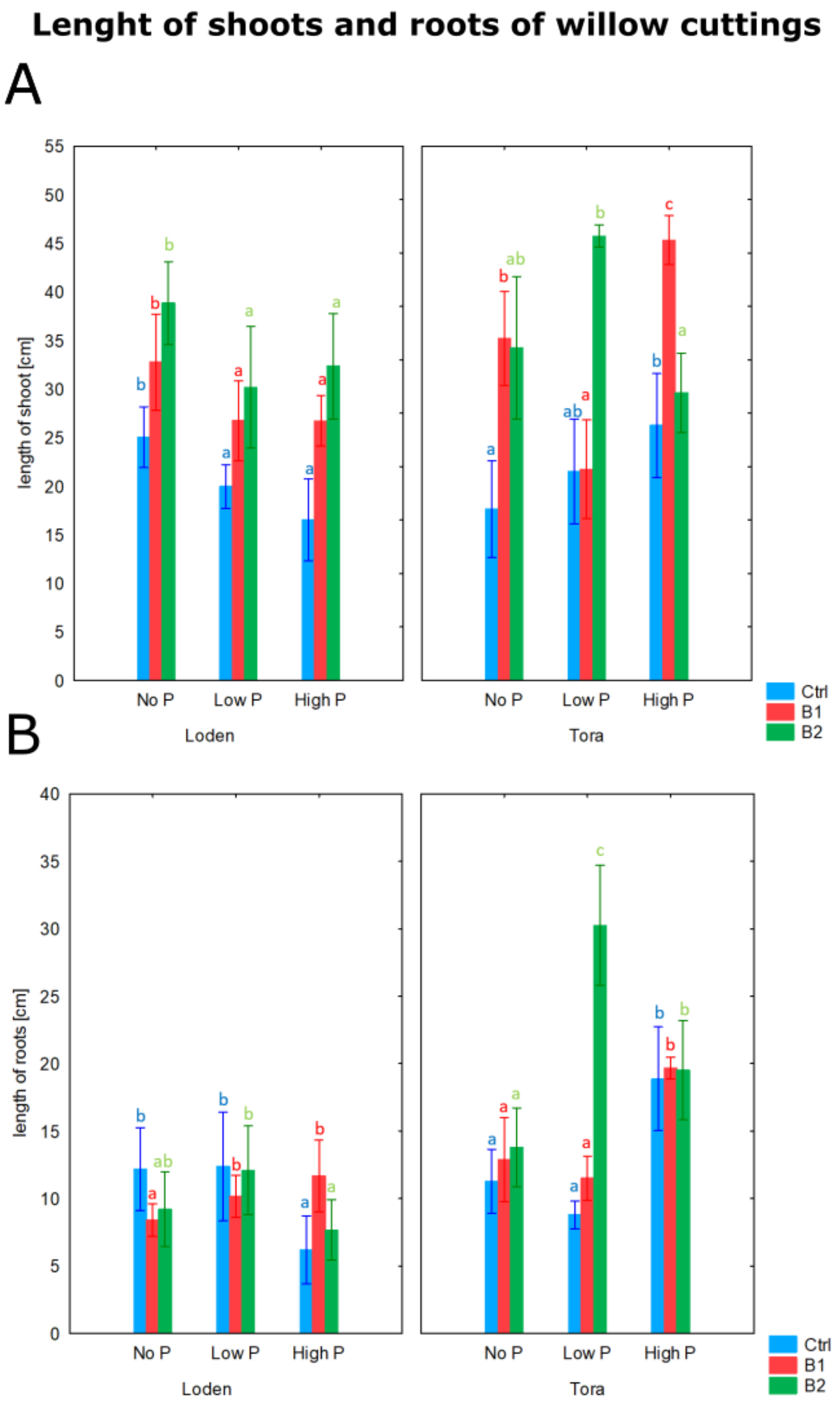

Figure 1 Length of shoots (A) and roots (B) in cm of willow cuttings from pot experiment after 5 weeks of cultivation in three different P concentrations (No P, Low P, High P). Plants were additionally inoculated with two bacterial strains B1 – red color, B2 – green color and were compared to control – blue color. Colored letters represent significant differences between P concentrations variants.

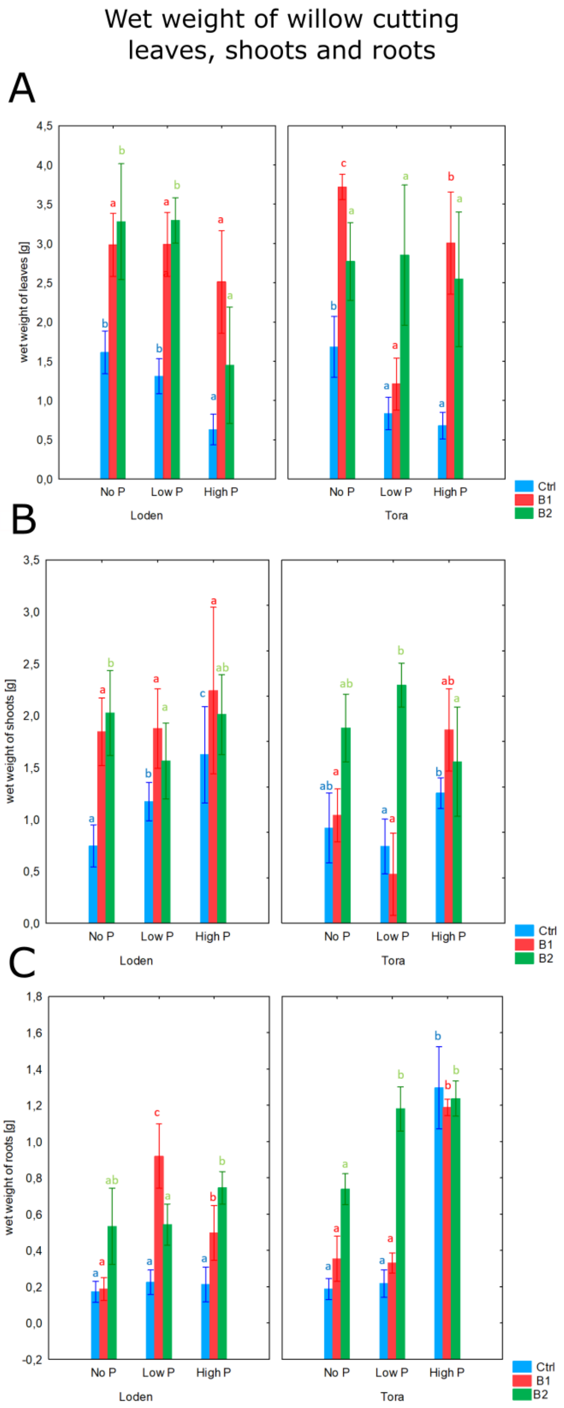

Figure 2 Weight of leaves (A) shoots (B) and roots (C) in g of willow cuttings from pot experiment after 5 weeks of cultivation in three different P concentrations (No P, Low P, High P). Plants were additionally inoculated with two bacterial strains B1 – red color, B2 – green color and were compared to control – blue color. Colored letters represent significant differences between P concentrations variants.

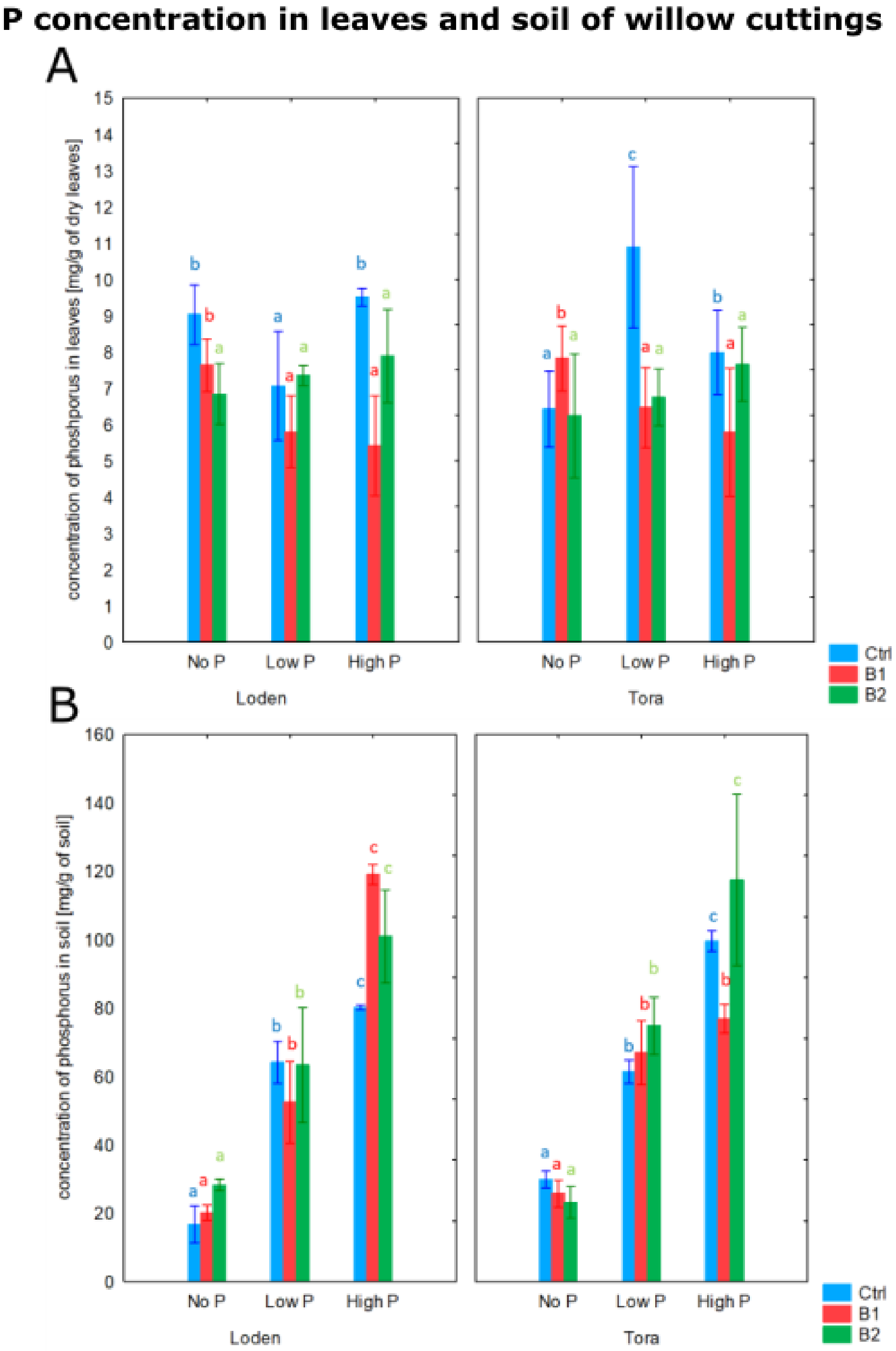

Figure 3 P content in leaves (A) soil (B) of willow cuttings from pot experiment after 5 weeks of cultivation in three different P concentrations (NoP, LowP, High P). Plants were additionally inoculated with two bacterial strains B1 – red color, B2 – green color and were compared to control – blue color. Colored letters represent significant differences between P concentrations variants.

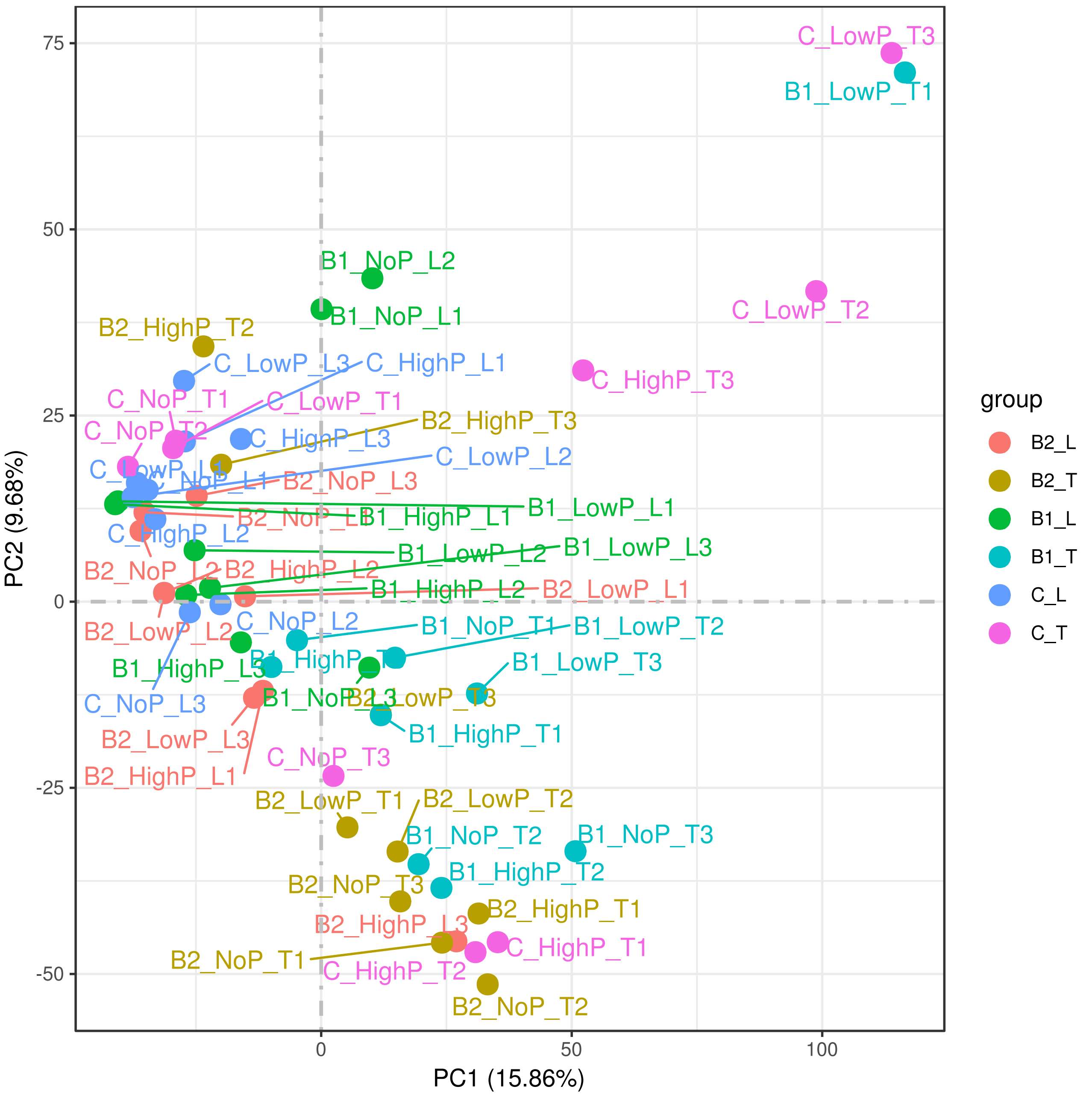

Figure 4 Principal component analysis (PCA) for all variants of experiment. Inoculation variants are denoted as follows: C – control; B1 – bacteria 1; B2 bacteria 2. Phosphate concentrations are donoted by NoP – no phosphate; LowP – low phosphate level (1M); HighP – high phosphate level (2M). Salix species used are detoned as L – Loden and T – Tora.

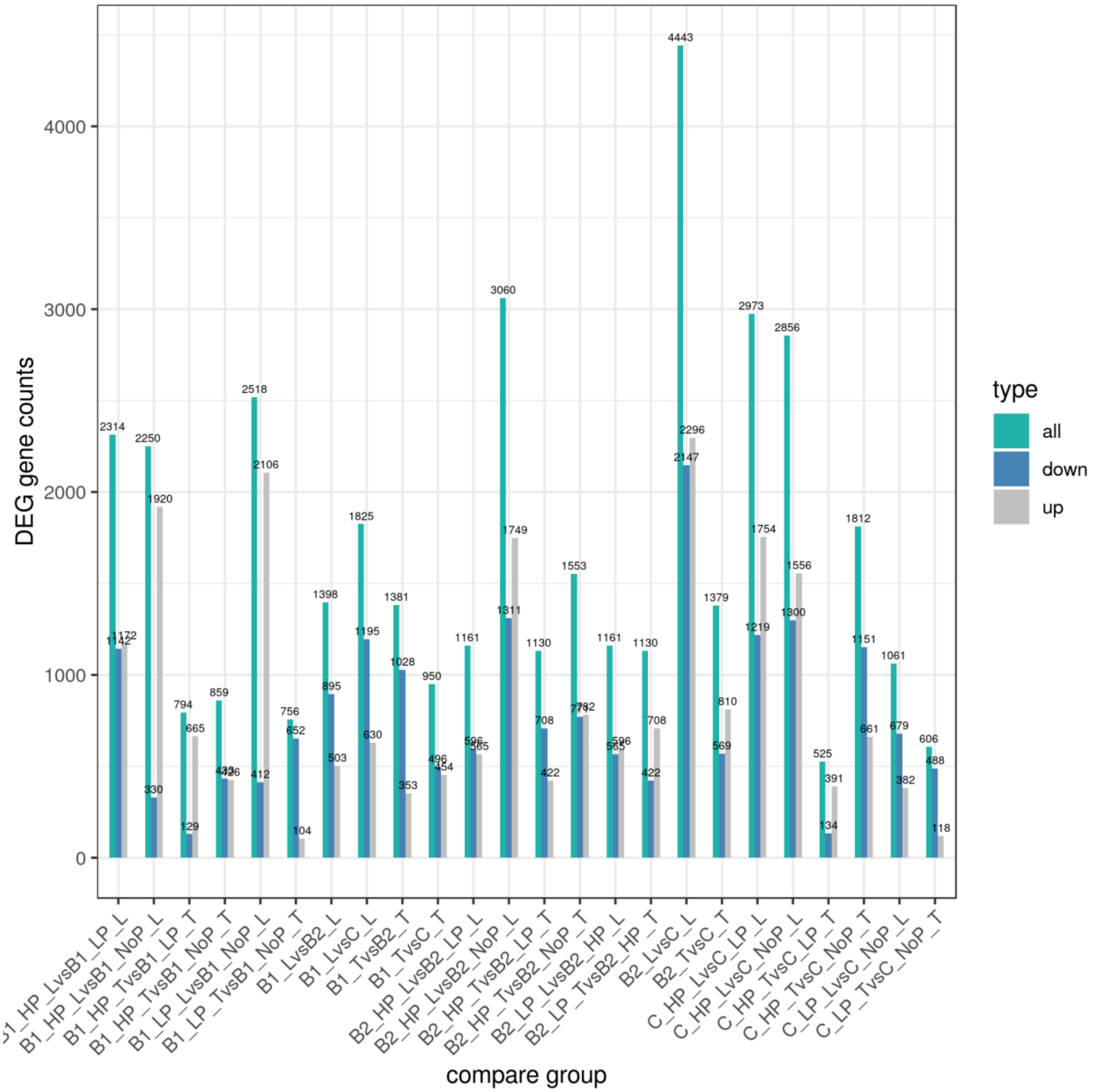

Figure 5 Gene count for each compared group represented in form of all, down regulated and up regulated genes. Second variant is always treated as reference. C –non inoluclated control; B1 – Bacteria 1; B2 – Bacteria 2; HP – High P; LP – Low P.
